# Thirty percent conversion efficiency from radiofrequency power to thrust energy in a magnetic nozzle plasma thruster

**DOI:** 10.1038/s41598-022-22789-7

**Published:** 2022-11-10

**Authors:** Kazunori Takahashi

**Affiliations:** 1 Department of Electrical Engineering, Tohoku University, Sendai, 980-8579 Japan; 2 Interdisciplinary Research Center for Non-equilibrium Plasma, Tohoku University, Sendai, 980-8579 Japan

**Keywords:** Plasma physics, Aerospace engineering

## Abstract

Innovations for terrestrial transportation technologies, e.g., cars, aircraft, and so on, have driven historical industries so far, and a similar breakthrough is now occurring in space owing to the successful development of electric propulsion devices such as gridded ion and Hall effect thrusters, where solar power is converted into the momentum of the propellant via acceleration of the ionized gases, resulting in a high specific impulse. A magnetic nozzle (MN) radiofrequency (rf) plasma thruster consisting of a low-pressure rf plasma source and a MN is an attractive candidate for a high-power electric propulsion device for spacecraft, as it will provide a long lifetime operation at a high-power level due to the absence of an electrode exposed to the plasma and a high thrust density. The high-density plasma produced in the source is transported along the magnetic field lines toward the open-source exit and the plasma is then spontaneously accelerated in the MN. By ejecting the plasma flow from the system, the reaction forces are exerted to the thruster structure including the source and the MN, and the spacecraft is resultantly propelled. The thruster will open the next door for space technologies, while the performance of the MN rf plasma thruster has been lower than those of the mature electric propulsion devices due to the energy loss to the physical walls. Here the thruster efficiency of about 30%, being the highest to date in this type of thruster, is successfully obtained in the MN rf plasma thruster by locating a cusp magnetic field inside the source, which acts as a virtual magnetic wall isolating the plasma from the source wall. The increase in the thrust by the cusp can be explained by considering the reductions of the loss area and the plasma volume in a thrust analysis combining a global source model and a one-dimensional MN model.

## Introduction

Electric propulsion (EP) is one of the advanced transportation technologies in space and plays an important role in space activities as a spacecraft engine in recent years^1–3^. The gaseous propellant is priorly ionized and the charged particles in the plasmas are energized and accelerated by applying electromagnetic fields. As electric power obtained by solar panels is converted into the kinetic energy of the propellant, the EP devices can provide a high specific impulse, which corresponds to a thrust per unit mass of the propellant, compared with chemical propulsion devices^4^. Representatively, EP devices such as gridded ion and Hall effect thrusters have been successfully used in various space missions, e.g., SMART-1 mission^5^, Dawn mission^6^, BepiColombo mission^7^, and Hayabusa 1 and 2 missions^8,9^. More recently, a new type of gridded ion thrusters utilizing iodine propellant has been successfully operated in orbit^10^. In these types of thrusters, the ions are electrostatically accelerated and exhausted together with the equal flux of the electrons supplied from neutralizers; zero net current exhausted from the system is maintained. When DC electric power is coupled with the plasmas, the electrodes in the thruster and the neutralizer have to be exposed to the plasmas and are often damaged by ion sputtering and thermal load. Therefore, the lifetime of the thruster has been a critical issue and been extended by elaborating designs of the gridded ion and Hall effect thrusters e.g., the 7-kW class NEXT ion thruster has been successfully operated for about 50,000 h on the ground test^11^. The lifetime extension would become a challenging problem when further increasing the operating power.

Some types of electrodeless plasma thrusters have been proposed and under investigation toward high-power, long-lived, and high-thrust-density EP devices, e.g., a variable specific impulse plasma rocket (VASIMR) and a MN radiofrequency (rf) plasma thruster, which is sometimes called a helicon thruster. In the former, most of the electric power is coupled with the ions via an ion cyclotron resonance heating (ICRH) and their perpendicular energy is converted into the directed axial energy by a magnetic nozzle (MN), where superconducting magnets are required to apply the strong magnetic fields for the ICRH^12^. For the latter case, most of the rf power is transferred to the electrons via a Joule and/or helicon wave heating processes, providing the high-density plasma production in the source, where the source can be operated with relatively low magnetic fields^13,14^. As the electron temperature is much larger than the ion temperature in the MN rf plasma thrusters, conversion processes from the electron energy to the thrust energy are key issues to improve the thruster performance.

When applying the MN to the rf plasma source, a number of experiments have shown that the electric fields are spontaneously formed and the ions in the high potential source are accelerated toward the low potential side^15^; the energetic electrons overcoming the potential drop neutralize the accelerated ions^16^. These observations have indicated that the MN rf plasma thruster does not require a neutralizer. The role of the spontaneous electrostatic acceleration is the conversion of the electron energy to the ion dynamic energy^17^. During the plasma expansion in the MN, an internal azimuthal plasma current spontaneously develops due to the diamagnetic nature; the axial Lorentz force arising from the diamagnetic current and the radial magnetic field increases the thrust, as predicted and demonstrated by theories and experiments^18–21^. The conversion efficiencies from the rf power to the thrust energy were about 0.5–5% in early experiments^22–26^ and approaches about 20% in recent years^27^, based on the scientific insights into the thrust generation processes. Some models and experiments have identified that the energy and momentum losses to the source wall yield the low thruster efficiency and the inhibition of the loss by increasing the strength of the axial magnetic fields have been discussed and demonstrated so far^28,29^. The efficiency of the thruster is still lower than the gridded ion and Hall effect thrusters, which is probably due to insufficient inhibition of the plasma loss to the wall; it is crucial to improve the thruster performance step-by-step.

Here we report a 30% conversion efficiency from the rf power to the thrust energy in the MN rf plasma thruster, which is the highest to date, where a cusp magnetic field is formed in the upstream side of the source tube, while maintaining the MN structure downstream of the source. It is clearly observed that the plasma inside the source tube is aligned along the cusp magnetic field; the upstream plasma is geometrically isolated from the source wall, resulting in the inhibition of the plasma loss to the wall and the improvement of the thruster performance. The detected thrust is consistent with the electron diamagnetic Lorentz force estimated from the radial plasma profile. The result is qualitatively explained by an analysis combining a global source model and a one-dimensional MN model, where changes in the effective loss area and the discharge volume are taken into account.

**Figure 1:**
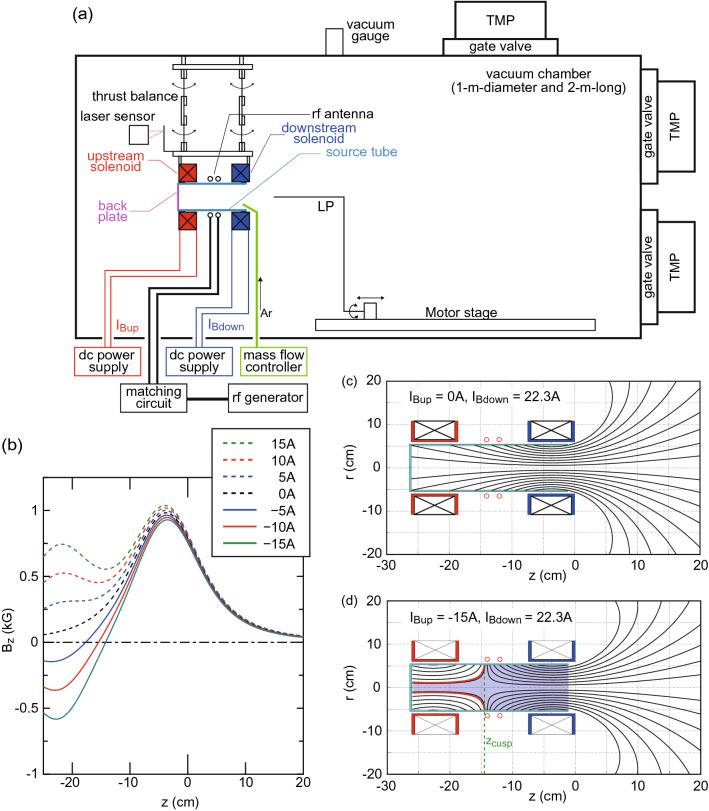
Experimental setup and magnetic field structures. (**a**) Schematic diagram of the experimental setup. (**b**) Calculated magnetic fields $B_z$ on the *z* axis for various upstream solenoid currents $I_{Bup}$, where the downstream solenoid current is maintained at $I_{Bdown}=22.3$ A. The two-dimensional profiles of the magnetic field lines for (**c**) $(I_{Bup}, \, I_{Bdown})=(0, \, 22.3 \, \mathrm{A})$ and (**d**) $(I_{Bup}, \, I_{Bdown})=(-15 \, \mathrm{A}, \, 22.3 \, \mathrm{A})$. The axial position giving the zero magnetic field on the radial center is defined as $z_{cusp}$ as drawn by the dotted line in (**d**). The bold red lines and the blue-colored regions in (**d**) show the field lines intersecting the inner source wall at $z=z_{cusp}$, and the volume for the global model analysis. The cusp is formed for $I_{Bup}<0$ and the axial position $z_{cusp}$ of the cusp can be shifted by changing $I_{Bup}$, while no drastic change is seen in the MN region.

## Experimental setup

The schematic of the experimental setup is shown in Fig.1a. The experiment is performed with the MN rf plasma thruster consisting of an 11-cm-outer-diameter, 10.5-cm-inner-diameter, and 25-cm-long glass source tube wound by a double-turn rf loop antenna centered at $z=-13$ cm and two solenoids centered at $z=-3.8\pm 0.2$ cm and $-22.5\pm 0.2$ cm, which are labeled as “downstream” and “upstream” solenoids, respectively. The axial position of the right-hand edge of the downstream solenoid holder is defined as $z=0$ and the open-source exit is set at $z=-1$ cm. The upstream side of the source tube is terminated by an insulator mica back plate. The whole structure of the thruster is attached to a pendulum thrust balance immersed in a 1-m-diameter and 2-m-long vacuum chamber evacuated to a base pressure less than $10^{-4}$ Pa by three turbomolecular pumping systems. As it has already been reported that the better thruster performance can be obtained when introducing the propellant gas near the open-source exit^30^, argon gas is introduced from the ceramic tube located near the thruster exit as seen in Fig.1a. The gas flow rate is maintained at 70 sccm (2.1 mg/s) and the pressure measured at the chamber sidewall is about 28 mPa, which is ten times higher than the standard background pressure recommended for testing gridded ion and Hall effect thrusters. This is due to the limited effective pumping speed (being about 4500 $\hbox {Ls}^{-1}$ for argon), while no detectable change in the thrust of the MN rf plasma thruster has been induced by the different pumping speeds giving the pressures of 28 mPa and 88 mPa^31^. DC solenoid currents $I_{Bup}$ and $I_{Bdown}$ are supplied to the upstream and downstream solenoids by dc power supplies, respectively, for applying the magnetic fields. Each solenoid has a copper wire would around the solenoid holder 638 turns (29 turns and 22 turns in the axial and radial directions). Figure 1b shows the calculated magnetic fields on the *z* axis for various $I_{Bup}$ and $I_{Bdown}=22.3$ A, where the positive and negative currents provide the magnetic fields directing rightward and leftward on their centers, respectively. Two-dimensional structures of the magnetic field lines for $(I_{Bup}, \, I_{Bdown})=(0, \, 22.3 \, \mathrm{A})$ and $(-15 \, \mathrm{A}, \, 22.3 \, \mathrm{A})$ are drawn in Fig. 1c and d, respectively. By supplying the negative current for $I_{Bup}$, it can be seen that the zero axial magnetic field on the axis, called the cusp magnetic field, is formed inside the source tube. The expanding magnetic field, i.e., the MN, is formed downstream of the source tube, and no drastic change can be seen in the MN region when changing $I_{Bup}$. The rf loop antenna is powered by a 13.56 MHz rf generator via an impedance matching box, where two variable capacitors in the matching box are tuned in advance to minimize the rf power reflection during the plasma production and no detectable power reflection can be seen for all the data. The plasma produced inside the source and expanding along the MN is visually confirmed.

The axial displacement of the pendulum is induced by the plasma production, which corresponds to the thrust, and is measured by a laser displacement sensor. The detailed procedure can be found in ‘Method’ section. The absolute value of the thrust is obtained by multiplying a calibration coefficient relating the displacement to the force (see ‘Method’ section). The thruster efficiency $\eta _T$ is estimated from the measured thrust *F*, the mass flow rate of the propellant ${\dot{m}}$, and the forward rf power $P_{rf}$ as

1 $$\begin{aligned} \eta _T= & {} \frac{F^2}{2 {\dot{m}} P_{rf}}, \end{aligned}$$

where the electric power for the solenoids is not taken into account here. Hence $\eta _T$ indicates the conversion efficiency from the rf power to the thrust energy. Furthermore, the rf power transfer efficiency $\eta _p$, which is defined as the ratio of the power absorbed by the plasma to the rf power, is also assessed by measuring the rf antenna current as described in ‘Method’ section, since it directly affects the plasma production and the resultant thrust generation. A 3-mm-diameter planar Langmuir probe (LP), which radially faces, is mounted on an axially and radially movable motor stage. The ion saturation current $I_{is}$ of the negatively biased LP is measured, which is given by $I_{is}=0.61 e n_p u_B S$ with the elementary charge *e*, the plasma density $n_p$, the Bohm velocity $u_B$, and the detection area *S* of the LP. Typical electron temperature measured at (*r*, *z*) = (0, 10 cm) is about $6\pm 1$ eV; the plasma density for $I_{is}=1$ mA can be roughly estimated as $3.8\pm 0.5 \times 10^{17} \, \mathrm{m^{-3}/mA}$. As the Bohm velocity is given by $u_B=(k_B T_e/m_i)^{1/2}$ with the Boltzmann constant $k_B$, the electron temperature $T_e$, and the ion mass $m_i$, $I_{is}$ is proportional to $n_p T_e^{1/2}$, being a rough indicator of the electron pressure ($p_e=n_pk_BT_e$) with an error of $T_e^{1/2}$.

**Figure 2:**
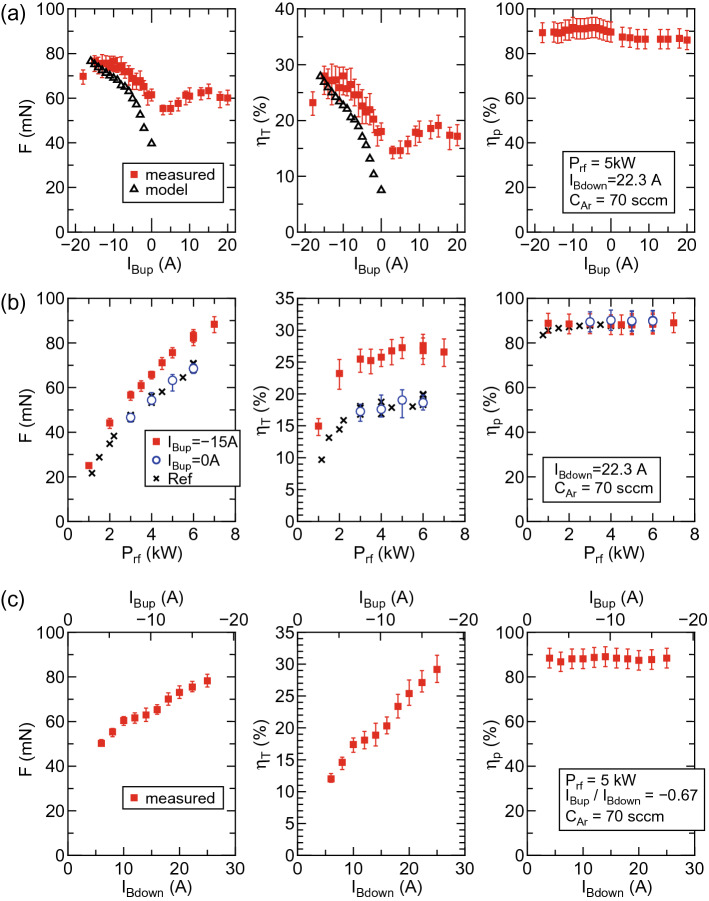
Characterized thruster performance. Measured thrust *F*, thruster efficiency $\eta _T$, and rf power transfer efficiency $\eta _p$ as functions of (**a**) $I_{Bup}$ for $I_{Bdown}=22.3$ A (filled red squares), (**b**) $P_{rf}$ for $I_{Bup}=0$ A (open blue circles) and for $I_{Bup}=-15$ A (filled red squares), and (**c**) $I_{Bup}$ and $I_{Bdown}$ with maintaining the constant ratio of $I_{Bup}/I_{Bdown}=-0.67$ (filled red squares). The data from Ref.^27^ is plotted by crosses in (**b**) for comparison. The open black triangles in (**a**) are obtained from the thruster model including the global source model and the one-dimensional MN model, where the changes in the plasma loss area and the volume are considered and $\eta _p=0.9$ is used for the calculation. The thruster performance (*F* and $\eta _T$) can be increased by forming the cusp in the source ($I_{Bup}<0$) as in (**a**, **b**) and by increasing the magnetic field strength as in (**c**), while the efficient rf power coupling of $\eta _p\sim 90\%$ is maintained for all the conditions. The maximum thruster efficiency of $\eta _T\sim 30\%$ is successfully obtained. The increase in the thrust can be qualitatively explained by the model as in Fig.1a.

## Results and discussion

Filled red squares in Fig. 2a show the measured thrust *F*, the thruster efficiency $\eta _T$, and the rf power transfer efficiency $\eta _p$ as functions of $I_{Bup}$ for $P_{rf}=5$ kW and $I_{Bdown}=22.3$ A. *F* and $\eta _T$ slightly increase for $I_{Bup}=10$-15 A, which would be due to the increase in the magnetic field strength inside the source (see Fig.1b) and the resultant inhibition of the plasma loss to the source wall as reported in Ref.^29^. Very interestingly, the significant increases in *F* and $\eta _T$ can be obtained for $I_{Bup}<0$ with the cusp inside the source, in spite of the decreases in the source field strength as shown in Fig. 1b. The thruster efficiency of about $\eta _T\sim 27.5 \%$ can be obtained in the range of $-15 \, \mathrm{A} \le I_{Bup} \le -10 \, \mathrm{A}$.

*F*, $\eta _T$, and $\eta _p$ as functions of $P_{rf}$ are assessed when powering only the downstream solenoid, i.e., $(I_{Bup}, \, I_{Bdown})=(0, \, 22.3 \, \mathrm{A})$, as plotted by open blue circles in Fig. 2b, and compared with the previous experiment (crosses)^27^. It should be noted that $I_{Bdown}=22.3$ A can provide the peak magnetic field strength being close to the previous experiment^27^. The differences between the open blue circles and crosses are only the source tube materials and sizes (10.5-cm-inner-diameter and 25-cm-long glass tube and 9.5-cm-inner-diameter and 20-cm-long ceramic tube for the present and previous experiments, respectively); the similar values of *F*, $\eta _T$, and $\eta _p$ are obtained. Red filled squares in Fig. 2b show the results for $(I_{Bup}, \, I_{Bdown})=(-15 \, \mathrm{A}, \, 22.3 \, \mathrm{A})$, indicating the performance improvement by forming the cusp inside the source over the rf power range tested here. Only the magnetic field strength can be controlled with the unchanged spatial structure of the magnetic field lines by changing both $I_{Bup}$ and $I_{Bdown}$ while maintaining $I_{Bup}/I_{Bdown}$ at a constant level. Figure 2c shows *F*, $\eta _T$, and $\eta _p$ as functions of $I_{Bup}$ and $I_{Bdown}$ under the condition of $I_{Bup}/I_{Bdown}=-0.67$. *F* and $\eta _T$ continuously increases with the increase in the field strength, being consistent with the earlier experiment^20^. For the maximum field strength case of ($I_{Bup}$, $I_{Bdown}$) = ($-16.8$ A, 25 A), the thruster efficiency of $\eta _T\sim 30 \%$, being the highest to date, can be successfully obtained. It is noted that $\eta _p$ is about 90% for all the data in Fig. 2. The measured total resistance $R_{total}$ including the antenna ($R_{vac}$) and plasma ($R_p$) resistances ranges from 4.5 to 6 $\Omega$, while the antenna resistance is about $0.56 \, \Omega$. Since the condition of $R_{total}\gg R_{vac}$ is maintained for all the conditions, the parametric change in $R_{total}$ within the range of 4.5–6.5 $\Omega$ does not significantly affect $\eta _p$. This fact shows that the power absorbed by the plasma is unchanged by the external parameters in the present experiment and implies that the enhanced thrust is not due to the change in the rf power coupling but due to the presence of the cusp.

**Figure 3:**
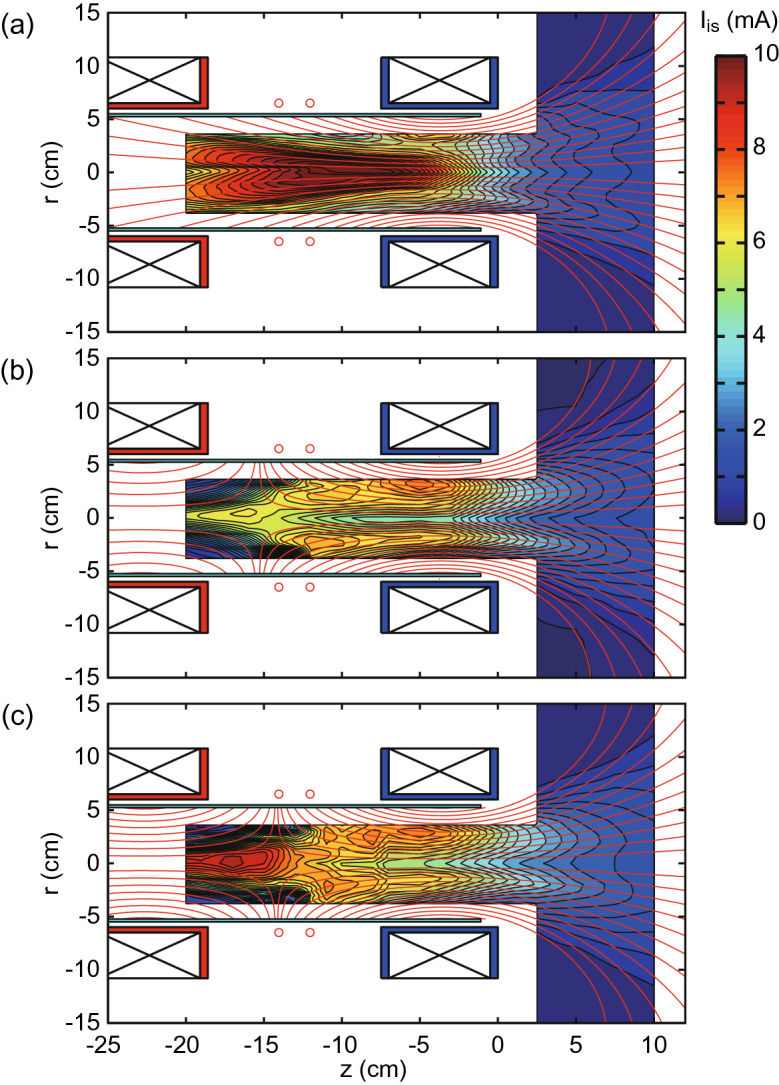
Two-dimensional plasma mapping inside the source. Two-dimensional profiles of the ion saturation current $I_{is}$ of the LP, which roughly mirrors the electron pressure profiles, for (**a**) $I_{Bup}=0$ A, (**b**) $I_{Bup}=-10$ A, and (**c**) $I_{Bup}=-15$ A, together with the calculated magnetic field lines, where the downstream solenoid current and the rf power are maintained at $I_{Bdown}=22.3$ A and $P_{rf}=5$ kW, respectively. The profiles roughly follow the magnetic field lines for all the cases. Especially, it can be found that the ion current close to zero is observed at the peripheral region upstream of the rf antenna as in (**b**, **c**). The presence of the cusp provides the geometric isolation of the plasma from the source wall.

**Figure 4:**
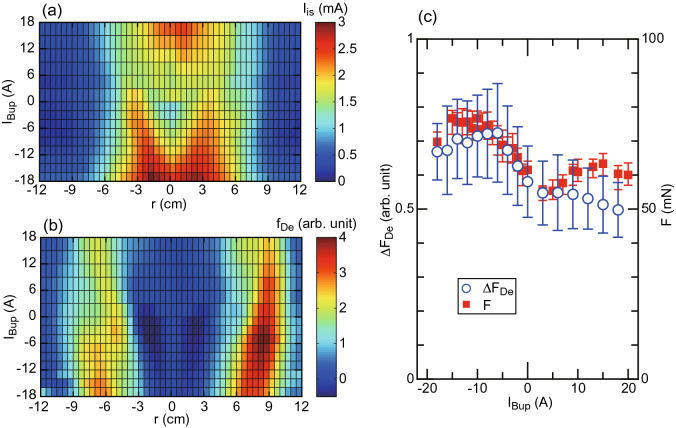
Qualitative estimation of the Lorentz force in the MN. Radial profiles of (**a**) the ion saturation current $I_{is}$ of the LP taken at $z=5$ cm and (**b**) the relative value of the local electron-diamagnetic Lorentz force $f_{De}$ calculated from the measured $I_{is}$ and the calculated magnetic fields. (**c**) The Lorentz force $\Delta F_{De} = 2\pi \int _0^{r} r f_{De} dr$ integrated over the cross section (blue open circles), together with the measured thrust *F* (filled red squares, same data as Fig.2a).

To understand the effect of the cusp, two-dimensional profiles of the ion saturation current $I_{is}$ are taken and shown in Fig. 3 for ($I_{Bup}$, $I_{Bdown}$) = (a) (0 A, 22.3 A), (b) ($-10$ A, 22.3 A), and (c) ($-15$ A, 22.3 A). The plasma follows the magnetic field lines and contact with the radial source wall upstream of the rf antenna for the no cusp case as in Fig.3a. When the cusp is formed inside the source as in Fig. 3b and c, the nearly zero density is observed in the peripheral region upstream of the cusp and the plasma is geometrically isolated from the wall. This effectively decreases the plasma loss area to the wall and the discharge volume, whose effect will be incorporated in the simple model described later. It is noted that the off-axis density peak appears inside the source and transported along the magnetic field lines as clearly seen in Fig. 3b and c. Such a structure has been observed in a number of experiments and discussed with the electron heating by the rf electromagnetic fields^32–36^; 2-D simulations of wave-plasma interactions have shown the off-axis profile of the rf electromagnetic fields^37,38^. The off-axis density peak is also reproduced in a 2-D particle-in-cell simulations^39,40^. Furthermore, a 2D simulation with the cusp in the source have been performed more recently^41^, showing the off-axis density profile and the reduction of the density near the wall upstream of the cusp.

The previous studies have shown that the Lorentz force due to the electron diamagnetic current and the radial magnetic field in the MN increases the thrust and is the major component of the total thrust. The local Lorentz force $f_{De}$ is then described as^20^

2 $$\begin{aligned} f_{De}= & {} -\frac{B_r}{B_z}\frac{\partial p_e}{\partial r} \propto -\frac{B_r}{B_z}\frac{\partial I_{is}}{\partial r}, \end{aligned}$$

where the ion saturation current $I_{is}$ is assumed to be proportional to the electron pressure. Figure 4a shows the radial profile of $I_{is}$ taken at $z=5$ cm as a function of $I_{Bup}$, indicating the center-peaked and annular profiles for $I_{Bup}>0$ and $I_{Bup}<0$, respectively. Figure 4b shows the local Lorentz force $f_{De}$ calculated by Eq. (2) with the measured $I_{is}$ and the calculated magnetic fields. It can be found that the Lorentz force is generated near the plasma edge where the pressure gradient is large. Assuming the axisymmetric profile of $f_{De}$, the Lorentz force $\Delta F_{De}$ integrated over the cross section is plotted by open circles in Fig. 4c, together with the measured thrust *F*, where the error bar for $\Delta F_{De}$ originates from the asymmetry between $r>0$ and $r<0$ in Fig. 4b. Since the calculated $\Delta F_{De}$ can qualitatively explain the variation in the thrust as seen here, it can be deduced that the increase in the Lorentz force contributes to the performance improvement. It should be noted that the actual thrust component due to the Lorentz force in the MN is obtained by the volume integration; only the qualitative discussion can be made by using $\Delta F_{De}$.

For further understanding of the cusp effect on the thruster performance, the changes in the plasma loss area and the volume are incorporated into a global source model connected to a one-dimensional MN model. The details of the model are described in ‘Method’ section. Briefly, the electron temperature $T_e$ and the plasma density $n_s$ in the source region are assumed to be uniform; the particle and power balance equations are numerically solved. As observed in Fig. 3b and c, the plasma is geometrically isolated from the source wall by the cusp, resulting in the reduction of the loss area and the discharge volume. It is assumed that the plasma upstream of the cusp is confined within the magnetic field lines (bold red lines in Fig. 1d) intersecting the inner source wall at $z=z_{cusp}$, where $z_{cusp}$ is the axial position giving the zero magnetic field on the radial center. It is assumed that the loss area is the back and radial walls where the plasma contacts and the discharge volume is the blue-colored region as shown in Fig. 1d. Substituting the plasma density and electron temperature in the source into the one-dimensional MN model and the flux conservation law, the axial profiles of the velocity and density can be obtained. Then the total thrust can be calculated from these physical quantities inside the source and in the MN. It should be emphasized again that the model can be used only to qualitatively explain the increase in the thrust by the cusp, since the loss area corresponding to the wall where the plasma contacts and the plasma volume do not significantly change for $I_{Bup}>0$.

**Figure 5:**
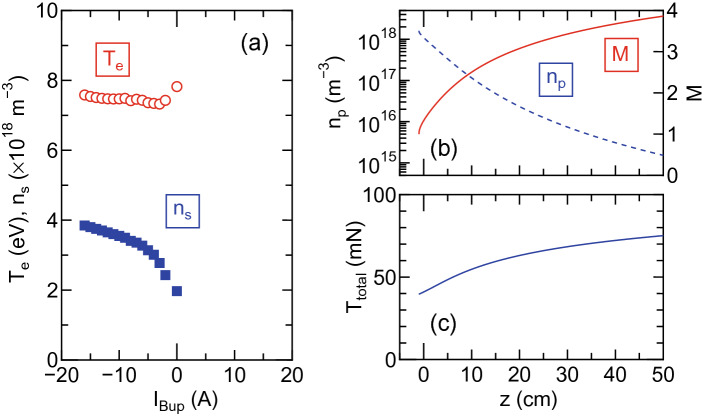
The results from the global source model and the one-dimensional MN model. (**a**) The plasma density $n_s$ (blue filled squares) and electron temperature $T_e$ (red open circles) in the source, as a function of $I_{Bup}$. Typical axial profiles of (**b**) the ion Mach number *M*, the plasma density $n_p$, and (**c**) the thrust $T_{total}$. The increase in the source plasma density for $I_{Bup}<0$ can be seen. The velocity increases and the plasma density decays along the MN. The increase in $T_{total}$ along the MN is confirmed. As the finally obtained thrust would depend on the axial location of the plasma detachment, which is still an open question, $T_{total}$ at $z=50$ cm is used for discussion and is plotted by open triangles in Fig. 2a.

Figure 5a shows $n_s$ and $T_e$ in the source as a function of $I_{Bup}$, where the axial position $z_{cusp}$ of the cusp is shifted by $I_{Bup}$, implying that the source density can be increased by supplying the larger negative current $I_{Bup}$ to the upstream solenoid, i.e., by forming the cusp closer to the rf antenna. In this calculation, the rf power transfer efficiency is maintained at $\eta _p=0.9$ as observed in Fig.2a. Figure 5b shows the typical axial profiles of the ion Mach number *M* and the plasma density $n_p$ in the MN; it shows the ion acceleration and the density decay along the MN. The total thrust, i.e., the axial momentum flux, is found to increase along the MN, where the finally obtained thrust would correspond to $T_{total}$ at the axial position where the plasma detachment occurs. As the plasma detachment is still an open question^42–46^, $T_{total}$ at $z=50$ cm is used for discussion. The calculated $T_{total}$ at $z=50$ cm and the thruster efficiency $\eta _T$ are plotted by open triangles in Fig. 2a, where the analysis is performed only for $I_{Bup}<0$ as no cusp reducing the loss area and the plasma volume is formed for $I_{Bup}>0$. It is found that the model qualitatively explains the performance improvement for $I_{Bup}<0$, i.e., by the cusp. This indicates that the cusp acts as the virtual wall isolating the plasma from the source wall and reducing the particle and energy losses to the physical walls.

**Figure 6:**
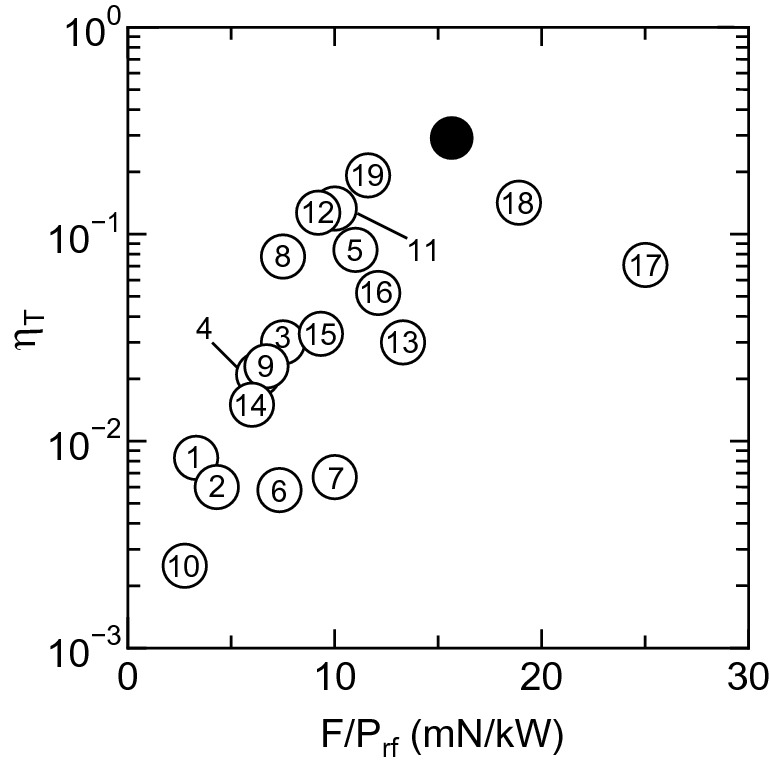
Thruster performances from literature and the present experiment. Thruster efficiency $\eta _T$ versus $F/P_{rf}$ from the published literature (open circles with the number corresponding to the indexes of the publication column in Table 1) and the present data showing the maximum efficiency (a black filled circle). This clearly shows the increase in the thruster efficiency compared with the previous studies.

As already described before, the thruster assessments have been performed with various designs and parameters; typical performance data from literature are summarized in Table 1. Figure 6 shows the thruster efficiency $\eta _T$ calculated from the measured thrust *F*, the rf power $P_{rf}$, and the mass flow rate ${\dot{m}}$ of the propellant, as a function of $F/P_{rf}$. The performance data in Table 1 are plotted by open circles in Fig. 6 with the number corresponding to the indexes labeled by the publication column in Table 1. The present data showing the maximum efficiency is also plotted by a filled circle in Fig. 6, clearly indicating the increase in the thruster efficiency by the presence of the cusp inside the source tube.

**Table 1:** Performances of the MN rf plasma thrusters from the literature.

Publications	$P_{rf}$	*F*	$F/P_{rf}$	$\eta$
	(kW)	(mN)	(mN/kW)	(%)
1-Takahashi et al. APL2011^23^	0.9	3	3.3	0.83
2-Pottinger et al. JPD2011^22^	0.65	2.8	4.3	0.6
3-Takahashi et al. PRL2011^47^	0.8	6	7.5	3.0
4-Charles et al. APL2012^48^	0.8	5	6.3	2.1
5-Takahashi et al. PRL2013^20^	1	11	11.0	8.4
6-Shabshelowitz and Gallimore JPP2013^24^	1.5	11	7.33	0.58
7-Williams and Walker JPP2013^25^	0.6	6	10	0.67
8-Takahashi et al. JPD2013^49^	2	15	7.5	7.8
9-Charles et al. APL2013^50^	0.9	6	6.7	2.3
10-Harle et al. PSST2013^51^	0.4	1.1	2.75	0.25
11-Takahashi et al. PSST2014^52^	2	20	10	13.3
12-Takahashi et al. PSST2015^53^	6	58	9.7	13.3
13-Kuwahara et al. JPP2017^54^	3	40	13.3	3.0
14-Oshio et al. IEPC2017^55^	1	6	6.0	1.5
15-Trezzolani et al. IEPC2017^56^	0.15	1.4	9.33	3.3
16-Trezzolani et al. IEPC2017^57^	0.07	0.85	12.1	5.2
17-Takahashi et al. JPP2020^58^	1	25	25	7.1
18-Navarro-Cavallé et al. IEPC2019^59^	0.45	8.5	18.9	14.2
19-Takahashi et al. SciRep2021^27^	6	69.63	11.6	19.24

It should be noted again that the thruster efficiency is calculated from the rf power $P_{rf}$ from the rf generator; hence it does not take the solenoid power and the power loss at the rf generator into account. The solenoids can be partially replaced by permanent magnets as investigated in previous studies^60–62^. Furthermore, developments of rf systems for the thrusters have also been progressed such as efficient switching-type rf generators^63,64^ and compact frequency-tunable impedance matching techniques^65,66^. The system developments providing less power losses at the solenoids and the rf generator, reducing the system size, and improving the controllability, remains further challenging issues toward a flight model of the MN rf plasma thruster.

Even if no electrode is exposed to the plasmas, the insulator source wall will be sputtered when the energy of the ions impinging the wall exceeds the threshold of the sputtering as being a crucial problem in the Hall effect thrusters, where the accelerated ions having an energy close to the discharge voltage would be the major factor for the wall erosion^67^. The ions impinging the source wall in the MN rf plasma thruster is only accelerated by a sheath at the wall, which has a voltage of about $5.2T_e$ for argon^68^. As discussed by Del Valle et al., the threshold energy of the sputtering for a quartz glass, being about 35 eV, is close to the typical sheath voltage in the low-pressure rf discharge^69^. Therefore, the wall erosion induced by the sputtering will be minimized by choosing the wall materials properly and by reducing the electron temperature near the wall (i.e., the sheath voltage). The lifetime of the MN rf plasma thruster has not been verified yet and remains further development issue.

## Conclusion

The thruster efficiency estimated from the measured thrust, the rf power, and the mass flow rate of the propellant, is successfully increased up to about thirty percent by forming the cusp magnetic field at the upstream region of the source. It is demonstrated that the cusp field geometrically isolates the plasma from the source wall, resulting in the increases of the thrust and the thruster efficiency. The performance improvement can be qualitatively understood by considering the reductions of the plasma loss area and the discharge volume inside the source, where the thruster analysis is made by combining the global source model and the one-dimensional magnetic nozzle model. The presently reported thruster efficiency is the highest to date in this type of thruster called the helicon thruster; the present results would lead to a new transportation technology in space, i.e., the high-power and long-lived electric propulsion device.

## Methods

### Thrust measurement procedure

**Figure 7:**
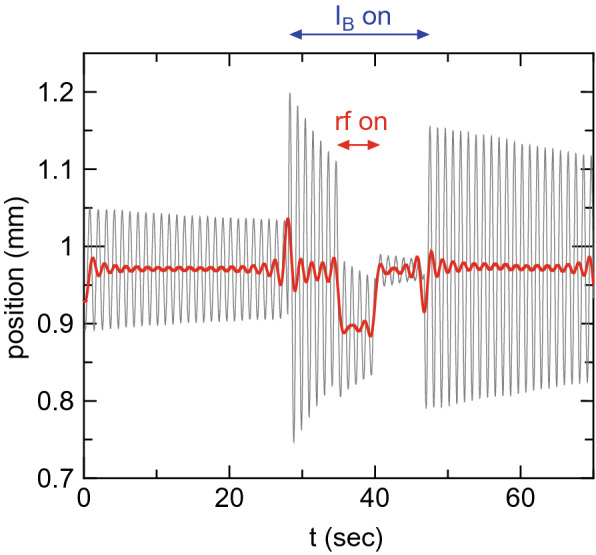
Thrust measurement procedure. Typical raw signal from the laser displacement sensor (a gray thin line), where the solenoid currents and the rf power are turned on for $t\sim 25--47$ s and $t\sim 35--40$ s, respectively. A red bold line shows the filtered signal for minimizing the amplitude of the pendulum oscillation and for estimation of the equilibrium positions for tuning on only the solenoid currents ($t\sim 27--35$ s) and for turning on both the solenoid currents and the rf power ($t\sim 35--40$ s).

Argon gas is continuously introduced into the thruster in advance. A gray line in Fig. 7 shows the raw signal from the laser displacement sensor, which contains the specific oscillation frequency of about 1 Hz due to the pendulum motion. The measured signal is converted into an amplitude spectrum by a Fast Fourier transform and filtered in the frequency domain. The filtered amplitude spectrum is converted into the temporal signal via a Inverse Fast Fourier transform as drawn by a red line in Fig. 7, minimizing the oscillation component of the pendulum.

After confirming a stable equilibrium position of the thrust balance ($t \sim$ 0–27 s), both the solenoid currents ($I_{Bup}$ and $I_{Bdown}$) are simultaneously turned on (at $t\sim$27 s in Fig. 7), where a rapid increase in the amplitude of the pendulum motion and slight change in the equilibrium position can be seen for $t \sim$ 27–35 s due to the inductions of the eddy current on the metallic materials and a magnetic force on magnetic materials (e.g., SUS304). At $t\sim 35$ s, the rf power is turned on for about 5 s, clearly showing the change in the equilibrium position for $t \sim$ 35–40 s by the plasma production. The laser sensor signal gets back to the value at $t \sim$ 27–35 s after turning off the rf power ($t\sim$ 40–47 s) and back to the initial equilibrium position after turning off the solenoid currents ($t>47$ s). The difference in the equilibrium positions for $t\sim$ 27–35 s (turning on only the solenoid currents) and $t\sim$ 35–40 s (turning on both the solenoid currents and the rf power) gives the displacement induced only by the plasma production, which does not contain the displacements induced by applying the magnetic field and by injecting the gas. The absolute value of the thrust can be obtained by multiplying the calibration coefficient described in the next section. Since the rf power is turned on only for 5 s in the present experiment, no significant thermal drift has been seen as in Fig. 7.

### Thrust balance calibration

**Figure 8:**
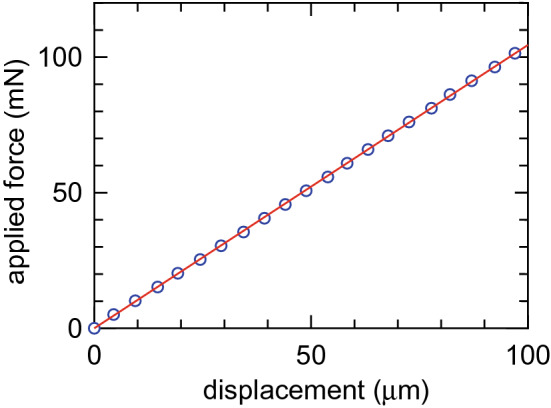
Thrust balance calibration. Measured displacement (blue open circles) versus applied force, together with a fitted linear line (a red solid line) giving the calibration coefficient.

Before pumping down the vacuum chamber, a calibration procedure is performed by applying known axial forces to the thruster attached to the balance and measuring the displacement. The measured displacement as a function of the applied force is plotted by blue open circles in Fig. 8. The linearity is well maintained over the force of less than 100 mN at least; the characteristic can be fitted by a linear line as drawn by a red sold line in Fig. 8. The fitted line gives the calibration coefficient as $\sim 1.045 \, \mathrm{mN}/\upmu {\text{m}}$. It is confirmed that the coefficient is unchanged after performing the experiment and venting the chamber.

### RF power transfer efficiency

The rf power transfer efficiency $\eta _p$ is defined as a ratio of the rf power absorbed by the plasma to the total rf power. A well-known equivalent circuit model is used here to estimate $\eta _p$^70^. Assuming that the input rf power is dissipated by the plasma resistance $R_p$ and the rf antenna resistance $R_{vac}$, the power transfer efficiency $\eta _p$ can be given by

3 $$\begin{aligned} \eta _p= & {} \frac{R_p}{R_{total}} = \frac{R_{total}-R_{vac}}{R_{total}}, \end{aligned}$$

where $R_{total}$ is the total resistance during the plasma production. These resistances can be estimated by measuring the rf antenna current by locating a Rogowski-type current sensor that can be used up to a frequency of 20 MHz (Pearson Electronics, Model 5046) and the net power of the rf generator, which simply corresponds to the forward power minus the reflected power. The antenna resistance of $R_{vac}\sim 0.56 \, \Omega$ is obtained via the same procedure with no gas injection and no plasmas. Since the rf antenna, the rf feedthrough, and the circuit components in the matching box, are water-cooled at a constant temperature of 20 degrees C, the antenna resistance $R_{vac}$ is unchanged during the experiment.

### Thruster model

An analysis combining a global source model and a one-dimensional MN model, which is used for the analyses of the open triangles in Fig. 2a and the data in Fig. 5, is described here. The plasma density $n_s$ and the electron temperature $T_e$ in the source are modeled from the power and particle balance equations in the global model assuming uniform profiles^68^. The balance equation between the particle generation by the ionization process and the loss from the system is given as

4 $$\begin{aligned} K_{iz} n_s n_g V = n_s u_B A_{eff}, \end{aligned}$$

where $K_{iz}$, $n_g$, V, and $A_{eff}$ are the ionization rate constant, the neutral density, the plasma volume, and effective plasma loss area, respectively. The description of $A_{eff}$ and the approximated expression of $K_{iz}$ can be found in Ref.^68^ with the radial ($h_R$) and axial ($h_L$) center-to-edge density ratios. By numerically solving Eq. (4), the electron temperature $T_e$ can be obtained for the given gas pressure and the source geometry. The power balance between the input energy and the energy lost from the system is given as

5 $$\begin{aligned} \eta _p (P_{rf}-P_{ref})= & {} e n_s A_{eff} u_B E_T, \end{aligned}$$

where $P_{rf}$ and $P_{ref}$ are the forward and reflected rf powers, respectively. In the present experiment, the reflected power $P_{ref}$ is undetectable for all the data. $E_T$ is the energy loss due to collisional processes and electron-ion pairs escaping from the system and can be written by

6 $$\begin{aligned} E_T= & {} E_i + E_e + E_c, \end{aligned}$$

7 $$\begin{aligned}&E_i = 5.2 T_e \,  \, \mathrm{(for \, argon)}, \end{aligned}$$

8 $$\begin{aligned}&E_e = 2 T_e,\end{aligned}$$

9 $$\begin{aligned}&E_c = E_{iz} + \frac{K_{exc}}{K_{iz}}E_{exc} + 3 \frac{m_e}{m_i}\frac{K_{el}}{K_{iz}}T_e, \end{aligned}$$

where $E_i$, $E_e$, $E_c$, $E_{iz}$, and $E_{exc}$ are the ion kinetic energy loss, the electron kinetic energy loss, the collisional energy loss, the ionization energy, and the excitation energy, respectively. In the calculation, the approximated expression of the ionization ($K_{iz}$), excitation ($K_{exc}$), and elastic scattering ($K_{el}$) rate constants, which are functions of the electron temperature $T_e$, are used^68^. Substituting $T_e$ obtained from Eq. (4) into Eq. (5), $n_s$ can be numerically calculated for a given neutral density. Although the propellant gas is introduced near the thruster exit in the present experiments, the simple gas model is considered here. When the gas is introduced into the source at a constant mass flow rate ${\dot{m}}$, the local neutral density $n_g$ can be estimated from ${\dot{m}} = m_g n_g v_g A_s$, where $m_g$, $v_g$, and $A_s$ are the neutral mass, the neutral velocity (assumed to be 400 $\mathrm{ms^{-1}}$), and the cross section of the source tube, respectively.

As already mentioned in the ‘Results and discussion’ section, the upstream plasma is geometrically isolated from the source wall when applying the cusp magnetic field, resulting in the reductions of the wall area where the plasma contacts and the plasma volume are effectively reduced. This is geometrically estimated from the magnetic field line intersecting the source inner wall at $z=z_{cusp}$ as drawn by the bold red lines and the blue-colored region in Fig. 1d, where the plasma is assumed to be confined within the bold red lines in the upstream region. These effects are taken into account by modifying the effective loss area $A_{eff}$ and the plasma volume *V* in Eq. (4, 5), where $A_{eff}$ corresponds to the area of the wall to which the plasma (the blue-colored region) contacts, and *V* is obtained by calculating the volume of the blue-colored region. As plotted in Fig. 5, the plasma density in the source increases for $I_{Bup}<0$ cases with the increase in $|I_{Bup}|$.

Once $n_s$ and $T_e$ in the source are obtained, the results are substituted into the one-dimensional MN model^71^. Briefly, the thrust is given by the sum of the electron pressure in the source and the Lorentz force exerted on the MN as

10 $$\begin{aligned} T_{total}= & {} n_s k_B T_e A_s - -\int _0^z \frac{n_p k_B T_e A}{B_z}\frac{\partial B_z}{\partial z'}dz', \end{aligned}$$

where the axial momentum lost to the radial wall, which has been detected when the plasmas are significantly lost to the wall^72^, does not appear in the one-dimensional model and $A_s$ is the cross section of the source. The axial velocity of the ions can be given by a similar expression with the physical nozzle model as

11 $$\begin{aligned} \frac{M^2 - M_i^2}{2} - \ln \left( \frac{M}{M_i}\right) = \ln \left( \frac{B_{zi}}{B_z}\right) , \end{aligned}$$

where *M* is the ion Mach number and the subscript *i* denotes the value to the MN entrance. To connect the global source model and the MN model, the plasma density and the ion Mach number at the MN entrance (i.e., at the open-source exit) are assumed to be $h_L n_s$ and $M_i=1$, respectively. The assumption of $M_i=1$ at the source exit and the MN entrance, which corresponds to an energy of half an electron temperature, requires a density decay by a factor of $\exp (-1/2)\sim 0.6$ according to the Boltzmann relation. This can be briefly validated by the density profiles in Fig. 3. Furthermore, a number of experiments have shown the appearance of the supersonic ions near the source exit^14,15^. The local plasma density $n_p$ in the MN can be given from the particle flux conservation along the MN and the magnetic flux conservation as^71,73^

12 $$\begin{aligned} n_p M u_B A= & {} h_L n_s M_i u_B A_s, \end{aligned}$$

13 $$\begin{aligned} B_z A= & {} B_{zi} A_s. \end{aligned}$$

By numerically solving Eqs. (4–13), the source plasma density $n_s$, the electron temperature $T_e$, the local plasma density $n_p$, the ion Mach number *M*, and the total thrust $T_{total}$ can be obtained. It is noted that the inner diameter (10.5 cm) and length (25 cm) of the source tube installed in the present experiment are used for all the calculation.

## Data Availability

The data that support the figures within this paper are available from corresponding author upon reasonable request.

## References

[1] Mozouffre S (2016). Electric propulsion for satellites and spacecraft: Established technologies and novel approaches. Plasma Sources Sci. Technol..

[2] Levchenko I, Xu S, Teel G, Mariotti D, Walker MLR, Keidar M (2018). Recent progress and perspectives of space electric propulsion systems based on smart nanomaterials. Nat. Commun..

[3] Levchenko I, Bazaka K, Ding Y, Raitses Y, Mazouffre S, Henning T, Klar PJ, Shinohara S, Schein J, Garrigues L, Kim M, Lev D, Taccogna F, Boswell RW, Charles C, Koizumi H, Shen Y, Scharlemann C, Keidar M, Xu S (2018). Space micropropulsion systems for Cubesats and small satellites: From proximate targets to furthermost frontiers. Appl. Phys. Rev..

[4] Goebel DM, Katz I (2008). Fundamentals Electric Propulsion: Ion and Hall Thrusters.

[5] Foing BH, Racca GD, Marini A, Evrard E, Stagnaro L, Almeida M, Koschny D, Frew D, Zender J, Heather J, Grande M, Huovelin J, Keller HU, Nathues A, Josset JL, Malkki A, Schmidt W, Noci G, Birkl R, Iess L, Sodnik Z, McManamon P (2006). SMART-1 mission to the moon: Status, first results and goals. Adv. Space Res..

[6] Brophy J, Russell C, Raymond C (2012). The Dawn ion propulsion system. The Dawn Mission to Minor Planets 4 Vesta and 1 Ceres.

[7] Steiger C, Montagnon E, Accomazzo A, Ferri P (2020). BepiColombo mission to Mercury: First year of flight. Acta Astronaut..

[8] Kuninaka H, Nishiyama K, Funaki I, Yamada T, Shimizu Y, Kawaguchi J (2007). Powered flight of electron cyclotron resonance ion engines on Hayabusa Explorer. J. Propul. Power.

[9] Nishiyama K, Hosoda S, Tsukizaki R, Kuninaka H (2020). In-flight operation of the Hayabusa2 ion engine system on its way to rendezvous with asteroid 162173 Ryugu. Acta Astronaut..

[10] Rafalskyi D, Martínez JM, Habl L, Rossi EZ, Proynov P, Boré A, Baret T, Poyet A, Lafleur T, Dudin S, Aanesland A (2021). In-orbit demonstration of an iodine electric propulsion system. Nature.

[11] https://www.nasa.gov/content/next-provides-lasting-propulsion-and-high-speeds-for-deep-space-missions.

[12] Bering EA, Chang-Díaz FR, Squire JP, Brukardt M, Glover TW, Bengtson RD, Jacobson VT, McCaskill GE, Cassady L (2008). Electromagnetic ion cyclotron resonance heating in the VASIMR. Adv. Space Res..

[13] Charles C (2009). Plasmas for spacecraft propulsion. J. Phys. D.

[14] Takahashi K (2019). Helicon-type radiofrequency plasma thrusters and magnetic plasma nozzles. Rev. Mod. Plasma Phys..

[15] Charles C (2007). A review of recent laboratory double layer experiments. Plasma Sources Sci. Technol..

[16] Takahashi K, Charles C, Boswell RW, Fujiwara T (2011). Electron energy distribution of a current-free double layer: Druyvesteyn theory and experiments. Phys. Rev. Lett..

[17] Fruchtman A (2006). Electric field in a double layer and the imparted momentum. Phys. Rev. Lett..

[18] Ahedo E, Merino M (2010). Two-dimensional supersonic plasma acceleration in a magnetic nozzle. Phys. Plasmas.

[19] Takahashi K, Lafleur T, Charles C, Alexander P, Boswell RW (2011). Electron diamagnetic effect on axial force in an expanding plasma: experiments and theory. Phys. Rev. Lett..

[20] Takahashi K, Charles C, Boswell RW (2013). Approaching the theoretical limit of diamagnetic-induced momentum in a rapidly diverging magnetic nozzle. Phys. Rev. Lett..

[21] Merino M, Ahedo E (2016). Effect of the plasma-induced magnetic field on a magnetic nozzle. Phys. Plasmas.

[22] Pottinger S, Lappas V, Charles C, Boswell R (2011). Performance characterization of a helicon double layer thruster using direct thrust measurements. J. Phys. D.

[23] Takahashi K, Lafleur T, Charles C, Alexander P, Boswell RW, Perren M, Laine R, Pottinger S, Lappas V, Harle T, Lamprou D (2011). Direct thrust measurement of a permanent magnet helicon double layer thruster. Appl. Phys. Lett..

[24] Shabshelowitz A, Gallimore AD (2013). Performance and probe measurements of a radio-frequency plasma thruster. J. Propul. Power.

[25] Williams LT, Walker MLR (2013). Thrust measurements of a radio frequency plasma source. J. Propul. Power.

[26] Furukawa T, Yarita Y, Aoyagi H, Nishida H (2022). Measurement of plasma flow and electron energy probability function in radio frequency plasma thruster with a magnetic cusp. J. Appl. Phys..

[27] Takahashi K (2021). Magnetic nozzle radiofrequency plasma thruster approaching twenty percent thruster efficiency. Sci. Rep..

[28] Lafleur T (2014). Helicon plasma thruster discharge model. Phys. Plasmas.

[29] Takahashi K, Sugawara T, Ando A (2020). Spatially- and vector-resolved momentum flux lost to a wall in a magnetic nozzle rf plasma thruster. Sci. Rep..

[30] Takahashi K, Takao Y, Ando A (2016). Modifications of plasma density profiles and thrust by neutral injection in a helicon plasma thruster. Appl. Phys. Lett..

[31] Takahashi K, Takao Y, Ando A (2019). Low-magnetic-field enhancement of thrust imparted by a stepped-diameter and downstream-gas-injected rf plasma thruster. Plasma Sources Sci. Technol..

[32] Takahashi K, Charles C, Boswell R, Cox W, Hatakeyama R (2009). Transport of energetic electrons in a magnetically expanding helicon double layer plasma. Appl. Phys. Lett..

[33] Charles C (2010). High density conics in a magnetically expanding helicon plasma. Appl. Phys. Lett..

[34] Ghosh S, Yadav S, Barada KK, Chattopadhyay PK, Ghosh J, Pal R, Bora D (2017). Formation of annular plasma downstream by magnetic aperture in the helicon experimental device. Phys. Plasmas.

[35] Gulbrandsen N, Fredriksen Å (2017). RFEA measurements of high-energy electrons in a helicon plasma device with expanding magnetic field. Front. Phys..

[36] Takahashi K, Akahoshi H, Charles C, Boswell RW, Ando A (2017). High temperature electrons exhausted from rf plasma sources along a magnetic nozzle. Phys. Plasmas.

[37] Tian B, Merino M, Ahedo E (2018). Two-dimensional plasma-wave interaction in an helicon plasma thruster with magnetic nozzle. Plasma Sources Sci. Technol..

[38] Jiménez B, Merino M, Ahedo E (2022). Wave propagation and absorption in a helicon plasma thruster and its plume. Plasma Sources Sci. Technol..

[39] Singh N, Rao S, Ranganath P (2013). Waves generated in the plasma plume of helicon magnetic nozzle. Phys. Plasmas.

[40] Emoto K, Takahashi K, Takao Y (2021). Axial momentum gains of ions and electrons in magnetic nozzle acceleration. Plasma Sources Sci. Technol..

[41] Ma X, Nishida H, Oshio Y, Furukawa T (2022). Numerical analysis of RF discharge in a nonuniform magnetic field. J. Appl. Phys..

[42] Arefiev AV, Breizman BN (2005). Magnetohydrodynamic scenario of plasma detachment in a magnetic nozzle. Phys. Plasmas.

[43] Merino M, Ahedo E (2014). Plasma detachment in a propulsive magnetic nozzle via ion demagnetization. Plasma Sources Sci. Technol..

[44] Olsen CS, Ballenger MG, Carter MD, Chang Díaz FR, Giambusso M, Glover TW, Ilin AV, Squire JP, Longmier BW, Bering EA, Cloutier PA (2015). Investigation of plasma detachment from a magnetic nozzle in the plume of the VX-200 magnetoplasma thruster. IEEE Trans. Plasma Sci..

[45] Takahashi K, Ando A (2017). Laboratory observation of a plasma-flow-state transition from diverging to stretching a magnetic nozzle. Phys. Rev. Lett..

[46] Little JM, Choueiri EY (2019). Electron demagnetization in a magnetically expanding plasma. Phys. Rev. Lett..

[47] Takahashi K, Lafleur K, Charles C, Alexander P, Boswell RW (2011). Electron diamagnetic effect on axial force in an expanding plasma: experiments and theory. Phys. Rev. Lett..

[48] Charles C, Takahashi K, Boswell RW (2012). Axial force imparted by a conical radiofrequency magneto-plasma thruster. Appl. Phys. Lett..

[49] Takahashi K, Charles C, Boswell R, Ando A (2013). Performance improvement of a permanent magnet helicon plasma thruster. J. Phys. D.

[50] Charles C, Boswell R, Takahashi K (2013). Boltzmann expansion in a radiofrequency conical helicon thruster operating in xenon and argon. Appl. Phys. Lett..

[51] Harle T, Pottinger SJ, Lappas VJ (2013). Helicon double layer thruster operation in a low magnetic field mode. Plasma Sources Sci. Technol..

[52] Takahashi K, Charles C, Boswell R, Ando A (2014). Effect of magnetic and physical nozzles on plasma thruster performance. Plasma Sources Sci. Technol..

[53] Takahashi K, Komuro A, Ando A (2015). Effect of source diameter on helicon plasma thruster performance and its high power operation. Plasma Sources Sci. Technol..

[54] Kuwahara D, Shinohara S, Yano K (2017). Thrust characteristics of high-density helicon plasma using argon and xenon gases. J. Propl. Power.

[55] Oshio, Y., Shimada, T. & Nishida, H. Experimental investigation of thrust performance on position relationship between RF antenna and magnetic cusp of RF plasma thruster. in *Proceedings of the 35th International Electric Propulsion Conference*, IEPC-2017-344 (2017).

[56] Trezzolani, F. *et al*. Development and test of an high power RF plasma thruster in project SAPERE-STRONG. in *Proceedings of the 35th International Electric Propulsion Conference*, IEPC-2017-462 (2017).

[57] Trezzolani, F. *et al*. Development and testing of a miniature helicon plasma thruster. in *Proceedings of the 35th International Electric Propulsion Conference*, IEPC-2017-519 (2017).

[58] Takahashi K, Takao Y, Ando A (2020). Increased thrust-to-power ratio of a stepped-diameter helicon plasma thruster with krypton propellant. J. Propul. Power.

[59] Navarro-Cavallé, J. *et al*. Development and characterization of the helicon plasma thruster prototype HPT05M. in *Proceedings of the 36th International Electric Propulsion Conference*, IEPC-2019-596 (2019).

[60] Takahashi K, Shida Y, Fujiwara T (2010). Magnetic-field-induced enhancement of ion beam energy in a magnetically expanding plasma using permanent magnets. Plasma Sources Sci. Technol..

[61] Virko VF, Virko YV, Slobodyan VM, Shamrai KP (2010). The effect of magnetic configuration on ion acceleration from a compact helicon source with permanent magnets. Plasma Sources Sci. Technol..

[62] Chen FF (2015). A compact permanent-magnet helicon thruster. IEEE Trans. Plasma Sci..

[63] Ziemba T, Euripides P, Slough J, Winglee R, Giersch L, Carscadden J, Schnackenberg T, Isley S (2006). Plasma characteristics of a high power helicon discharge. Plasma Sources Sci. Technol..

[64] Liang W (2018). An integrated RF power delivery and plasma micro-thruster system for nano-satellites. Front. Phys..

[65] Charles C, Boswell RW, Bish A (2013). Variable frequency matching to a radiofrequency source immersed in vacuum. J. Phys. D.

[66] Takahashi K, Imai R, Hanaoka K (2021). Automatically controlled frequency-tunable rf plasma thruster: Ion beam and thrust measurements. Front. Phys..

[67] Brown NP, Walker MLR (2020). Review of plasma-induced Hall thruster erosion. Appl. Sci..

[68] Lieberman MA, Lichtenberg AJ (2005). Principles of Plasma Discharges and Materials Processing.

[69] Del Valle JI, Chang Diaz FR, Granados VH (2022). Plasma-surface interactions with helicon plasma sources. Front. Phys..

[70] Lafleur T, Charles C, Boswell RW (2011). Characterization of a helicon plasma source in low diverging magnetic fields. J. Phys. D.

[71] Fruchtman A, Takahashi K, Charles C, Boswell RW (2012). A magnetic nozzle calculation of the force on a plasma. Phys. Plasmas.

[72] Takahashi K, Chiba A, Komuro A, Ando A (2015). Axial momentum lost to a lateral wall of a helicon plasma source. Phys. Rev. Lett..

[73] Smolyakov AI, Sabo A, Yushmanov P, Putvinskii S (2021). On quasineutral plasma flow in the magnetic nozzle. Phys. Plasmas.

